# Polydrug Use and Heterogeneity in HIV Risk Among People Who Inject Drugs in Estonia and Russia: A Latent Class Analysis

**DOI:** 10.1007/s10461-017-1836-0

**Published:** 2017-07-11

**Authors:** Isabel Tavitian-Exley, Marie-Claude Boily, Robert Heimer, Anneli Uusküla, Olga Levina, Mathieu Maheu-Giroux

**Affiliations:** 1 Department of Infectious Disease Epidemiology, Imperial College London, Norfolk place, London, W21PG UK; 2 Epidemiology of Microbial Diseases, School of Public Health, Yale University, New Haven, USA; 3 Faculty of Medicine, University of Tartu, Tartu, Estonia; 4 NGO Stellit, St Petersburg, Russian Federation; 5 Department of Epidemiology, Biostatistics and Occupational Health, McGill University, Montréal, Canada

**Keywords:** HIV, Risk behaviours, People who inject drugs, Polydrug use, Heroin/opioid, Amphetamines, Eastern Europe, Latent class analysis

## Abstract

Non-medical drug injection is a major risk factor for HIV infection in Russia and Estonia. Multiple drug use (polydrug) has further been associated with increased harms. We compared HIV, injecting and sexual risk associated with polydrug use among people who injected drugs (PWID) in 2012–2013 in Kohtla-Järve (Estonia, n = 591) and St Petersburg (Russia, n = 811). Using latent class analysis, we identified five (poly)drug classes, the largest consisting of single-drug injectors among whom an opioid was the sole drug injected (56% of PWID). The four remaining polydrug classes included *polydrug*-*polyroute injectors* who injected and used opiates and stimulants (9%), *opiate*-*stimulant poly*-*injectors* who injected amphetamine-type-stimulants with a primary opiate (7%) and *opiate*-*opioid poly*-*injectors* who injected opioids and opiates (16%). *Non*-*injection stimulant co*-*users* were injectors who also used non-injection stimulants (12%). In multivariable multinomial regressions, all four polydrug classes were associated with greater injection risks than single-drug injection, while *opiate*-*stimulant* and *opiate*-*opioid poly*-*injection* were also associated with having multiple sex partners. Riskier behaviours among polydrug-injectors suggest increased potential for transmission of blood-borne and sexually-transmitted infections. In addition to needles/syringes provision, services tailored to PWID drug and risk profiles, could consider drug-appropriate treatment and sexual risk reduction strategies to curb HIV transmission.

**Electronic supplementary material:**

The online version of this article (doi:10.1007/s10461-017-1836-0) contains supplementary material, which is available to authorized users.

## Resumen

La inyección de drogas no médicas es un factor principal de riesgo para la infección por el VIH en Rusia y Estonia. Además, el uso de múltiple drogas (poli-drogas) se ha asociado con un aumento de daños. Comparamos el estado de infección de VIH, y los comportamientos del riesgo de inyección y sexual asociado con el uso de poli-drogas entre las personas que inyectan drogas (PID) en 2012–2013 en Kohtla-Järve (Estonia, n = 591) y San Petersburgo (Rusia, n = 811). Utilizando el análisis de la clase latente, identificamos cinco clases de uso de (poli)drogas, el más grande constituido por inyectores de una solo droga, inyectando un único opioide (56%). Las cuatro clases restantes incluyeron inyectores de *poli*-*drogas/poli*-*rutas* que inyectaron y usaron opiáceos y estimulantes (9%), *poli*-*inyectores de opiáceo*-*estimulante* que inyectaron estimulantes de tipo anfetamina con un opiáceo primario (7%) y *poli*-*inyectores de opiáceo*-*opioides* que inyectaron opiáceos y opioides (16%). Los *co*-*usuarios de estimulantes no inyectables* eran inyectores que también usaban estimulantes no inyectables (12%). En las regresiones multinomiales multivariables, las cuatro clases de uso de poli-droga se asociaron con mayores riesgos de inyección que la inyección de un solo fármaco, mientras que la *poli*-*inyección de opiáceo*-*estimulante* y la *poli*-*inyección de opiáceos*-*opioides* también se asociaron con tener múltiples parejas sexuales. Comportamientos más riesgosos entre los inyectores de poli-drogas sugieren un mayor potencial para la transmisión de infecciones transmitidas sexual y por la sangre. Además de la provisión de agujas y jeringas, los servicios adaptados a los perfiles de riesgo en las PID podrían considerar incluir estrategias de tratamiento y de reducción de riesgo de transmisión sexual adecuadas para los usadores de diferentes drogas, para reducir la transmisión del VIH.

## Introduction

Non-medical drug injection has been a driver of HIV and hepatitis C (HCV) epidemics in Eastern Europe and central Asia, where people who inject drugs (PWID) were estimated to account for half of new HIV and a majority of HCV infections in 2014 [1–5]. Estonia and Russia in particular have reported some of the highest HIV prevalence in key populations–populations disproportionately affected by HIV—outside of sub-Saharan Africa [1, 6–11]. HIV sero-positivity among PWID was 53% in Tallinn, the capital of Estonia, and 70% in Kohtla-Järve, the fifth largest city in the country in 2007 [12–14]. In the Russian Federation, HIV prevalence ranged from 9% to 64% among PWID and was 59% among PWID in St Petersburg in 2009 [15, 16].

Kohtla-Järve and St Petersburg have epidemiologically comparable epidemics of drug use and HIV. Both cities are situated on the northern part of two major heroin trafficking corridors linking Afghanistan to the heroin markets of Western Europe [3, 17]. Although a majority of PWID in the region inject heroin or synthetic opioids (e.g. fentanyl, methadone) [16, 18, 19], Estonia reported one of the highest prevalence of amphetamine-type stimulant (ATS) use in Europe [15, 20]. Data further suggest that ATS has become a major secondary drug among PWID in Kohtla-Järve and St Petersburg [18, 21–23]. Polydrug use consists of the concurrent use of multiple illicit drugs and such practices have recently been facilitated by increased drug availability. These trends are worrisome as polydrug use among PWID has been associated with a greater risk of injury, infection, overdose and drug-induced deaths in many parts of the world [10, 24–26]. Stimulants have been associated with greater injecting and sexual risks, and are known to adversely affect drug treatment outcomes and adherence to antiretroviral therapy among PWID [24, 27–29]. Combining stimulants with opioids, for example, may lead to riskier behaviours and HIV infection among PWID, with potentially important implications for prevention and treatment programmes [29–31]. However, evidence on polydrug use and HIV risk remains limited and no epidemiological studies have examined the extent to which heterogeneity in drug injection among PWID in Eastern Europe is related to risk behaviours and prevalence of HIV infection [32–36].

Uncovering patterns of polydrug use can be challenging. Latent class analysis (LCA) has been used to empirically identify classes of individuals, based on a set of observed characteristics [37, 38]. So far, most LCA studies have been conducted among PWID who injected heroin and cocaine in the United States and Canada, and few of them have examined the relationship between polydrug use and HIV risk behaviours outside the North American context [39–41]. In our study, polydrug use was defined as injecting a main illicit drug and injecting or using one or more additional illicit substance (excluding cannabis or alcohol). Our study objectives were (i) to empirically identify classes of polydrug use on the basis of drug class and administration route, and (ii) to investigate whether injecting and sexual risk behaviours, demographic factors, HIV and HCV are associated with different categories of (poly)drug use among PWID.

## Methods

### Study Population

PWID were recruited between May and July 2012 in Kohtla-Järve and from November 2012 to June 2013 in St Petersburg, using respondent driven sampling (RDS), a variant of chain referral sampling [42–44]. Comparable recruitment criteria (men and women aged 18 or over, “having injected drugs in the past 30 days”, lived in St Petersburg or Kohtla-Järve and had provided informed consent for the study), survey methodology and questionnaires were used, details of which have been previously published [10, 16, 23, 45–47]. In brief, six seeds representing diverse PWID sub-groups in terms of gender, main drug used, age and HIV status, were selected through outreach programmes in Kohtla-Järve, and 16 seeds in St Petersburg. Each seed recruited up to three PWID from their personal network, who on completing the survey recruited a maximum of three new participants [10, 44, 48]. Recruitment was tapered once the HIV outcome converged to a sample equilibrium and the target sample size was reached [43]. The samples were recruited over 11 waves in Kohtla-Järve and 12 waves in St Petersburg.

### Measures

The study questionnaire for both sites included standardised items from established tools including the WHO Drug Injecting study Phase II survey (v2b) for risk behaviours [10, 49]. Information on social and demographics, injection and sexual risk behaviours, testing and access to harm reduction services was elicited in a structured confidential face-to-face interview using a questionnaire administered by trained fieldworkers.

The variables examined for association with polydrug classes included past month injecting risk, past 6 months sexual risk behaviour and serological markers for HIV, HCV and Herpes Simplex Virus-2 (HSV-2) infection. Key behavioural variables predictive of HIV and viral hepatitis transmission were examined: injecting frequency (≥daily vs. <daily), injecting intensity (≥2 day vs. <2 day on last day injected), sharing needles and syringes, sharing drug paraphernalia, back-loading (filling a syringe from another working syringe), multiple sex partners, having a sex partner who injected drugs and having been paid for sex [50]. The variable “any sex in the last 6 months” was used to exclude non-sexually active PWID when examining associations. Demographic and contextual variables included age, sex, ethnicity (non-Russian/ethnic Russian), living arrangements (unstable/stable), source of income (non-regular/salaried), contact with needle and syringe programme (NSP), past year drug substitution treatment (OST) and city (Kohtla-Järve/St Petersburg). HIV sero-status was assessed with HIV antigen/antibody combo-assay (ADVIA-Centaur, Siemens healthcare diagnostics) and HIV-I/II Score line immunoassay confirmatory test (INNO-LIA^®^, Fujirebio Europe) in Kohtla-Järve. Rapid oral HIV-I/II antibody tests were used in St Petersburg (OraQuick Advance^®^, OraSure Technologies Inc.) and confirmed at the City AIDS Centre [10]. HCV and HSV reactivity were measured in Kohtla-Järve only, using commercially available anti-HCV (Murex v4.0) and HSV-2 IgG ELISA kits (IBL International GmbH).

### Statistical Analyses

LCA was used to identify PWID subgroups with similar patterns in primary (main), and additional drug(s) injected or used (i.e. polydrug classes) [51]. LCA is a form of latent variable modelling which aims to identify underlying relationships in a defined set of observed variables to divide a heterogeneous population into more homogenous subgroups (latent classes), by grouping observations that display similar response patterns on these variables [37, 38]. LCA methodology is particularly useful and was selected above other methods such as cluster analysis or factor analysis for its ability to generate model-based class characterisations with conditional probabilities.

Seven variables of interest describing characteristics of polydrug use in the past month were used in our LCA. These included the main drug class injected (ATS or opiate/opioid), injection of additional opiate/opioid, injection of additional stimulant, use of additional opiate/opioid, use of additional stimulant, number of drugs injected and number of non-injection drugs used (Supplementary material, Figure S1). The seven drug use variables were entered into a latent class model and fitted to the data, starting with one class and progressively increasing the number of classes to six.

The selection of the best model was informed by several fit statistics, current epidemiology of drug use, meaningfulness and practical implications of classes. The fit statistics considered were Pearson’s Chi squared and Log likelihood ratio tests (LR), Lo-Mendell-Rubin likelihood ratio test for nested models (LRMT) and the Akaike (AIC) and Bayesian information criteria (BIC) [38, 52, 53]. The two RDS samples were jointly analysed, based on similarities in primary drugs injected, frequency of polydrug use and our objective of identifying common drug combinations across settings (Table S1). A dummy variable for city was included in the model as a covariate. That is, city effects were adjusted for in the LCA, but city was not included alongside the seven variables forming the latent classes. The LCA assumption of conditional independence was ascertained by examining bivariate residuals for each set of variables in LCA [54–56]. LCA was performed using Mplus version 7.4 [55].

Socio-demographic, programme and HIV risk behaviour variables were then compared between emergent sub-types of the best fit latent class model, in univariate and multivariate multinomial logistic regression [57, 58]. Pearson’s Chi squared test for categorical variables and Wald test p-values for coefficients in multinomial regression (i.e. log odds of each polydrug class) were derived. Multivariable models were adjusted for demographic (age, sex, education, ethnicity, income) and contextual variables (contact with NSP, city) based on a priori knowledge. The latent class with the largest membership was used as the referent category in order to maximise statistical power.

Multinomial logistic regression was performed using robust variance estimation to take into account the survey design, using the svy command in Stata version 13.1 [59]. The potential correlation of observations within the recruitment chains of RDS sampling was accounted for by clustering the standard errors within each recruitment seed [58]. RDS weights were not used as RDS weights did not influence weighted estimates when compared to unweighted estimates (Table S1) [60, 61]. Possible effect modification between behavioural risk and city were explored in multinomial regression. A complete case analysis was used and ten observations with missing data were disregarded. Models including biomarkers for HCV and HSV only included participants from Kohtla-Järve as these biomarkers were not collected in St Petersburg. RDS results are presented following guidelines outlined in STROBE-RDS [62].

### Ethics

Ethical approval was obtained from the *Ethics Review Board* of the University of Tartu (Estonia), the *Institutional Review Board* at NGO Stellit in St. Petersburg (Russian Federation) and the *Human Investigation Committee* at Yale University (USA).

## Results

Our study included 1402 active PWID who had injected drugs in the previous 4 weeks, were 18 years or older and lived in Kohtla-Järve (n = 591) or St Petersburg (n = 811). Sample characteristics were previously described [10, 16, 45–47] and are summarised in Table S1. RDS recruitment measures are shown in Table S2. Most PWID were male (76%), of Russian ethnicity (90%), had completed basic education (i.e. up to 9th grade) (68%) and injected for over 5 years (93%). Almost half had a non-regular income (47%) and 38% were under 30 years old. Past month contact with an NSP was 43% with more PWID reporting contact in Kohtla-Järve than in St Petersburg (Table S1).

### Latent Class Model and Polydrug Use Class Membership

Latent class models with 2, 3, 4, 5 and 6 classes were fitted to the data and fit statistics compared. Based on the different model fit indices and entropy, the five-class model was selected. Pearson’s Chi squared and Likelihood ratio tests suggested a better fit for model 5 although AIC and BIC statistics were marginally lower for model 6 (Table S3).The 5-class model was preferred after examination of the 5-class and 6-class models. Both produced similar class assignments and the two very small classes in model 6 limited interpretation and subsequent analyses (not shown).

**Fig. 1:**
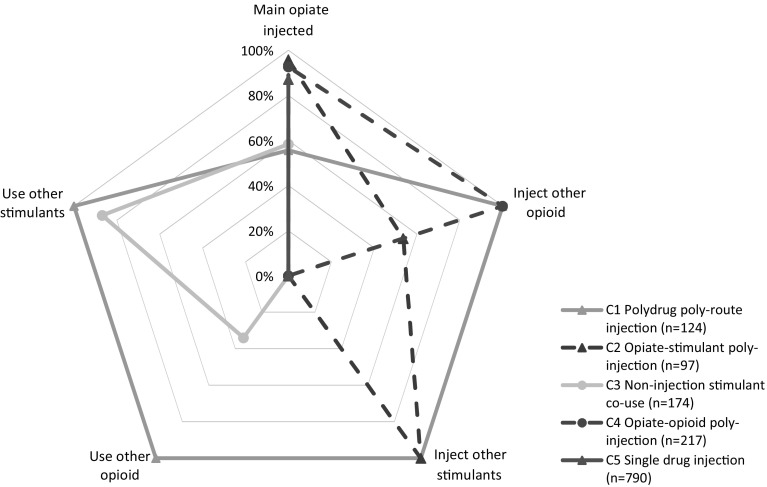
Polydrug use profiles for five-class solution of Latent Class model among PWID. The estimated probabilities for past month drug use are graphed based on latent class (C1–C5) membership shown in Table 1. The main axis (0–100%) shows the *probability of past month use* for each drug variable. For example *opiate*-*stimulant injectors* (C2) had a high probability of injecting an opiate as main drug (96%), medium probability of injecting another opiate (54%) and 100% probability of also injecting stimulants. The five binary variables only are shown for clarity (not shown are the variables for “number of drugs injected” and “number of non-injection drugs used”)

The largest class (class 5) included 56% (n = 790) of all PWID and the sizes of the four remaining classes ranged from 97 to 217 injectors (Table 1). Table 1 shows the conditional probabilities of endorsing a drug variable for an individual classified in their most likely class in the five-class model. Most class-specific response probabilities for binary indicators were above 0.70 or below 0.30, suggesting similar item responses for individuals in the same class and thus within-class homogeneity [52]. Drug use characteristics of each class in the 5-class model, including their qualitatively different primary (main) and additional drug class combinations are shown in Fig. 1.

**Table 1:** Conditional probabilities of drug use characteristics by class membership (5-class model)

Latent class model	Class 1 Polydrug poly-route injection (%, n)		Class 2 Opiate-stimulant poly-injection (%, n)		Class 3 Non-injection stimulant co-use (%, n)		Class 4 Opiate-opioid poly-injection (%, n)		Class 5 Single drug injection (%, n)		All classes (%, n)	
Class probabilities	9%	124	7%	97	12%	174	16%	217	56%	790	100%	1402
Primary drug injected^a^												
Primarily injected opiates^b^	56%	69	94%	91	58%	102	93%	201	87%	689	82%	1152
Primarily injected ATS	27%	33	4%	4	42%	73	6%	13	13%	102	16%	223
No primary drug	17%	22	2%	2	0%	0	1%	3	0%	0	2%	27
Other drugs and route of administration												
Injected other opiate/opioid	100%	41	54%	52	0%	0	100%	217	0%	0	23%	310
Injected other stimulant(s)^c^	100%	107	100%	97	0%	0	0%	0	0%	0	15%	204
Other opiate/opioid (non-injection)^d^	100%	52	0%	0	34%	59	0%	0	0%	0	8%	111
Other stimulant (non-injection)	100%	104	0%	0	87%	151	0%	0	0%	0	19%	255
Number other drug(s) injected												
One	64%	79	46%	45	0%	0	97%	211	0%	0	24%	335
Two	30%	37	45%	44	0%	0	3%	6	0%	0	6%	87
Three	6%	8	8%	8	0%	0	0%	0	0%	0	1%	16
City^a^												
St Petersburg	34%	42	76%	74	8%	14	94%	205	60%	476	58%	811
Kohtla-Järve	66%	82	24%	23	92%	160	6%	12	40%	314	42%	591

Conditional probabilities are the probability that a PWID is a polydrug user, conditional on their answer “yes” to a specific drug question (i.e. observed variable). Conditional probabilities are graphed in Fig. 1 for each class of the five-class model

*ATS* Amphetamine-Type Stimulants

^a^Column totals

^b^Heroin/synthetic opioids

^c^Other stimulants included methamphetamines, ecstasy, cocaine and ketamine

^d^Non-injected use may be smoked, snorted or ingested in tablet or liquid form

The 124 PWID in class 1 had 56% probability of injecting a primary opiate drug and high probabilities of all other polydrug use variables (i.e. injection and use); a class referred to as *“polydrug polyroute injection”*. The 97 class 2 PWID had high probabilities of (i) injecting opiates as main drug (94%) and (ii) injecting additional stimulant(s) (100%) and (iii) a null probability of using other drugs; this class was referred to as *“opiate*-*stimulant poly*-*injection”.* Class 3 consisted of 174 PWID with similar probabilities of injecting opiates or ATS as primary drug (58% and 42%, respectively), a high probability of *using* additional stimulants (87%) and a null probability of *injecting* other drugs; class 3 was identified as *“non*-*injection stimulant co*-*use”*. The 217 class 4 PWID, referred to as “*opiate*-*opioid poly*-*injectors*”, were characterised by primary and secondary opiate/opioid injection (94% and 100%, respectively) and a null probability of *using* other drugs. Finally, class 5 PWID did not use or inject multiple drugs (“*single drug injection*”) and had 87% probability of injecting an opiate as main drug and null probability on all other (poly)drug indicators.

The distribution of class membership varied between sites with *“polydrug polyroute injection”* (class 1) and *“non*-*injection stimulant co*-*use”* (class 3) injectors having a higher probability of being from Kohtla-Järve (66% and 92%, respectively); *“opiate*-*stimulant poly*-*injection”* (class 2) *and* “*opiate*-*opioid poly*-*injection”* (class 4) were more likely to be from St Petersburg (76% and 94%, respectively). *Single drug injectors* (class 5) had a higher probability of being from St Petersburg (60%) than Kohtla-Järve (40%).

### Correlates of Polydrug Class Membership

Multinomial univariate comparisons of socio-demographic characteristics, injecting and sexual risk behaviours, HIV and HCV prevalence between polydrug classes are shown in Table 2. Polydrug classes differed significantly from the sample average (in 17 of 23 variables) and among them, *non*-*injection stimulant co*-*users* (class 3) differed most often in four demographic, seven drug risk and three sexual risk variables. *Opiate*-*stimulant poly*-*injectors* (class 2) and *opiate*-*opioid poly*-*injectors* (class 4) reported higher injecting and sexual risk behaviours than single-drug injectors but did not differ on demographic characteristics. Polydrug polyroute injectors (class 1) differed on two demographic and two injecting behaviours.

**Table 2:** Univariate comparisons of socio-demographics, service characteristics and HIV risk behaviours across latent classes

	All PWID (n = 1402)^a^		Class 1 Polydrug poly-route injection (n = 124)		Class 2 Opiate-stimulant poly-injection (n = 97)		Class 3 Non-injection stimulant co-use (n = 174)		Class 4 Opiate-opioid poly-injection (n = 217)		Class 5 Single drug injection (n = 790)		Pearson’s X^2^	X^2^ p-value^b^
Demographic characteristics														
Female gender	24%	335	28%	35	23%	22	31%	54	21%	45	23%	179	8.26	0.082
Age <30 years	38%	535	**45%** ^**c**^	**56**	41%	40	**52%** ^**c**^	**90**	36%	77	34%	272	21.8	0.000
Completed secondary school	26%	359	27%	34	26%	25	**25%** ^**c**^	**43**	22%	47	27%	210	19.4	0.013
Completed higher education	7%	96	3%	4	6%	6	1%	2	11%	23	8%	61		
Non-Russian	10%	145	**19%** ^**c**^	**23**	5%	5	**17%** ^**c**^	**30**	7%	14	9%	73	25.3	0.000
Non-regular income	47%	657	48%	59	46%	45	**29%** ^**c**^	**49**	48%	105	51%	399	28.5	0.000
City (Kohtla-Järve)	42%	591	66%	82	24%	23	92%	160	6%	12	40%	314	340.9	0.000
Service characteristics^d^														
Drug/substitution treatment	12%	161	5%	6	10%	10	15%	26	12%	25	12%	94	7.7	0.103
Contact with NSP	43%	570	54%	62	**28%** ^**c**^	**26**	**77%** ^**c**^	**125**	**19%** ^**c**^	**40**	44%	317	143.9	0.000
Injecting risk behaviours (last month)														
Injecting < 5 years	7%	96	**11%** ^**c**^	**13**	4%	4	**14%** ^**c**^	**24**	2%	5	6%	50	24.2	0.000
Injecting daily or more	31%	427	42%	52	**53%** ^**c**^	**51**	**20%** ^**c**^	**34**	**37%** ^**c**^	**81**	27%	209	50.7	0.000
Injected ≥ twice a day	46%	642	**61%** ^**c**^	**76**	**70%** ^**c**^	**68**	43%	74	**50%** ^**c**^	**107**	40%	317	47.1	0.000
Shared needles/syringes	36%	502	31%	38	**55%** ^**c**^	**53**	**11%** ^**c**^	**19**	**57%** ^**c**^	**122**	34%	270	105.3	0.000
Lent needles/syringes	37%	520	32%	39	**57%** ^**c**^	**55**	**15%** ^**c**^	**25**	**53%** ^**c**^	**114**	37%	287	78.3	0.000
Shared drug paraphernalia	42%	594	35%	43	**58%** ^**c**^	**56**	**16%** ^**c**^	**27**	**64%** ^**c**^	**139**	42%	329	105.7	0.000
Filled from working syringe	33%	462	33%	41	**47%** ^**c**^	**46**	**13%** ^**c**^	**23**	40%	86	34%	266	44.4	0.000
Sexual risk behaviours (last 6 months)														
Any sex in last 6 months	78%	1092	81%	100	**87%** ^**c**^	**84**	**85%** ^**c**^	**146**	74%	160	76%	602	13.0	0.011
≥2 sex partners	43%	447	42%	36	**54%** ^**c**^	**45**	**32%** ^**c**^	**43**	**54%** ^**c**^	**87**	41%	236	20.8	0.000
Regular sex partner injects	56%	438	60%	42	67%	33	**57%** ^**c**^	**70**	**73%** ^**c**^	**63**	51%	230	18.3	0.001
Casual sex partner injects	58%	218	63%	27	66%	27	52%	24	**78%** ^**c**^	**46**	50%	94	16.3	0.003
Ever paid for sex	5%	54	9%	8	4%	3	6%	8	2%	3	6%	32	4.7	0.319
Serological markers^e^														
HIV test positive	58%	818	53%	66	61%	59	60%	105	59%	127	58%	461	1.87	0.759
HCV reactive	74%	441	72%	59	65%	15	73%	116	75%	9	77%	242	2.75	0.599
HSV positive	32%	185	31%	24	39%	9	27%	43	50%	6	34%	103	4.28	0.368

^a^Column percentage

^b^Chi-square test

^c^Unadjusted multinomial regression coefficient p-values (statistically significant results (*p* < 0.05) are bolded). Pairwise comparisons using Class 5 as reference category

^d^Drug/substitution treatment in past 12 months refers to drug substitution in Kohtla-Järve and any treatment in St Petersburg. Needle/syringe programme (NSP) contact in last 6 weeks

^e^Serological markers for hepatitis C (HCV) and herpes simplex virus (HSV) were available for Kohtla-Järve only

More *polydrug polyroute injectors* (class 1) were younger than 30 years and of non-Russian ethnicity than single-drug injectors (class 5), injected more frequently and intensely. After adjusting for age, sex, education, income, ethnicity, contact with NSP and city in multivariable analysis, *polydrug polyroute injectors* had statistically significantly higher odds of injecting more frequently and intensely, sharing needles and syringes, drug paraphernalia and back-loading (filling a syringe from a pre-filled syringe) than single-drug injectors (Table 3).

**Table 3:** Adjusted multinomial analysis of demographic, injecting and sexual risk behaviours, and serological markers of infections with poly(drug) use latent class membership

	Adjusted odds ratios (95% CI)			
Reference group: Class 5 single drug injection	Class 1 Polydrug polyroute injection	Class 2 Opiate-stimulant poly-injection	Class 3 Non-injection stimulant co-use	Class 4 Opiate-opioid poly-injection
Demographic and services				
Female gender	1.1 (0.7–2.0)	0.8 (0.4–1.7)	1.4 (0.9–2.1)	0.8 (0.5–1.2)
Age <30	1.3 (0.9–1.8)	1.7 (1.0–2.9)	1.5 (1.0–2.2)	1.6 (1.1–2.3)^a^
Non-Russian	1.8 (1.1–3.1)^a^	0.8 (0.3–2.1)	1.1 (0.6–1.9)	1.6 (1.1–2.4)^a^
Completed secondary school	0.8 (0.5–1.3)	0.9 (0.5–1.6)	0.7 (0.5–1.0)^c^	0.9 (0.5–1.5)
Non-regular income	1.0 (0.7–1.6)	0.7 (0.4–1.2)	0.6 (0.5–0.7)^c^	0.8 (0.5–1.1)
City (Kohtla-Järve)	3.4 (1.7–6.6)^a^	0.5 (0.3–1.0)	14.9 (7.8–28.9)^c^	0.1 (0.1–0.2)^a^
Service characteristics^b^				
Contact with NSP (last 6 weeks)	0.7 (0.4–1.3)	0.7 (0.4–1.3)	1.0 (0.6–1.6)	0.9 (0.6–1.3)
Drug treatment (last 12 months)	0.3 (0.1–1.5)	0.4 (0.2–1.6)	0.7 (0.3–1.5)	0.7 (0.2–2.3)
Injecting risk behaviours (last month)				
Injecting <5 years	1.1 (0.7–1.9)	1.6 (0.4–6.3)	0.7 (0.4–1.3)	1.8 (0.5–5.9)
Injected daily or more	2.5 (1.1–5.7)^a^	3.0 (1.5–5.8)^a^	0.9 (0.5–1.5)	1.3 (1.0–1.8)
Injected ≥ twice a day	2.7 (1.3–5.9)^a^	4.0 (2.3–6.9)^a^	1.1 (0.8–1.6)	1.6 (1.1–2.4)^a^
Shared needles/syringes	2.5 (1.3–4.8)^a^	2.3 (1.7–3.2)^a^	1.6 (0.7–4.0)	1.6 (1.1–2.4)^a^
Lent needles/syringes	1.0 (0.5–1.9)	2.4 (1.4–3.9)^a^	0.9 (0.5–1.7)	1.4 (0.9–1.9)
Shared paraphernalia	2.7 (1.4–4.9)^a^	1.8 (1.2–2.7)^a^	2.4 (0.9–6.9)	1.2 (0.9–1.7)
Filled from working syringe	3.6 (2.3–5.8)^a^	1.8 (1.1–3.1)^a^	3.2 (1.4–7.2)^a^	0.7 (0.5–1.1)
Sexual risk behaviours (last 6 months)				
Any sex in last 6 months	1.1 (0.6–2.2)	1.9 (1.1–3.5)^a^	1.5 (1.1–2.2)^a^	0.9 (0.6–1.1)
≥2 sex partners	1.2 (0.8–1.8)	1.7 (1.2–2.4)^a^	1.1 (0.8–1.5)	1.6 (1.2–2.1)^a^
Regular sex partner injects	1.5 (0.8–3.2)	1.9 (0.8–4.4)	1.2 (0.9–1.6)	3.2 (2.1–4.9)^a^
Casual sex partner injects	1.7 (1.0–2.9)	1.3 (0.5–3.5)	1.1 (0.5–2.6)	2.1 (1.1–3.9)^a^
Ever paid for sex	1.0 (0.4–2.4)	0.6 (0.2–1.7)	0.5 (0.2–1.2)	0.5 (0.1–1.9)
Serological markers^c^				
HIV test positive	0.7 (0.4–1.3)	1.1 (0.7–1.8)	0.9 (0.7–1.3)	1.3 (0.9–1.8)
HCV reactive	0.7 (0.2–2.3)	0.6 (0.1–2.9)	0.8 (0.5–1.3)	0.8 (0.2–4.2)
HSV positive	0.6 (0.3–1.1)	1.1 (0.5–2.8)	0.6 (0.4–1.0)	2.0 (0.6–6.6)

Multivariable multinomial regression models adjusted for age, sex, education, income, ethnicity, contact with needle and syringe programme and city (drug/substitution treatment did not differ significantly across classes)

*CI* confidence intervals, *NSP* needle and syringe programme

^a^Regression coefficient *p* value ≤0.05

^b^Drug/substitution treatment in past 12 months refers to drug substitution in Kohtla-Järve and any treatment in St Petersburg

^c^Serological markers for hepatitis C (HCV) and herpes simplex virus (HSV) were available for Kohtla-Järve only

*Opiate*-*stimulant poly*-*injectors* (class 2) had lower contact with an NSP, they reported more frequent injecting, greater injection intensity, more sharing and lending of needles and syringes, and back-loading in the past month than single-drug injectors (Table 2). More PWID in this class also reported multiple sex partners. After adjustment in multivariable analyses, *opiate*-*stimulant poly*-*injection* remained positively associated with frequent and intense injecting, sharing needles/syringes, sharing drug paraphernalia and back-loading compared to single drug injection (Table 3). *Opiate*-*stimulant poly*-*injectors* were also more likely to report any sex in the past 6 months and multiple sex partners than single-drug injectors.

More *non*-*injection stimulant co*-*users (*class 3) were under 30 years of age, of non-Russian ethnicity and from Kohtla-Järve and fewer reported an irregular source of income compared to single-drug injectors (Table 2). *Non*-*injection stimulant co*-*users* reported lower injection risk behaviours but more sex in the past 6 months compared to single-drug injectors and more PWID in this class had a regular partner who injected drugs. In multivariable analysis, *non*-*injection stimulant co*-*users,* who were less likely to have completed secondary education, to have a regular income and to be from Kohtla-Järve, reported generally lower injecting risks than single-drug injectors (Table 3). *Non*-*injection stimulant co*-*users* had greater odds of back-loading and were more likely to have had sex in the last 6 months compared to single-drug injectors.

The 217 *opiate*-*opioid poly*-*injectors* (class 4) reported more sharing of needles and syringes and more sharing of drug paraphernalia than single-drug injectors in unadjusted analysis (Table 2). More PWID in this class reported multiple sex partners and a sex partner who injected drugs compared to single-drug injectors. In multivariable analyses, *opiate*-*opioid poly*-*injectors* had higher odds of being younger than 30 years old, non-Russian and from St Petersburg (Table 3). *Opiate*-*opioid poly*-*injectors* had greater odds of injecting more intensely and of sharing needles/syringes, compared to single-drug injectors. They were also more likely to have multiple sex partners and a sex partner who injected drugs.

Despite significant differences in injecting and sexual risk behaviours, no statistically significant differences were found in HIV and HCV prevalence when any of the polydrug classes were compared to single drug-injectors, who were mainly opiate injectors. Differences in the prevalence of HIV or HCV among classes may emerge using another reference group. For example, *opiate*-*opioid poly*-*injectors* were more likely to be HIV positive compared to *polydrug*-*polyroute injectors* (adjusted OR 1.8; 95% confidence interval (CI) 1.1–3.2).

Potential effect modification between behavioural risk and city, examined in multinomial regressions, suggested that odds ratios were consistent for sexual and injecting risk behaviours, except for injecting frequency. The odds of frequent injecting remained significantly higher for poly-injectors (compared to single-drug injectors) in St Petersburg but was not significant in Kohtla-Järve in models examining effect modification (Table S4).

## Discussion

Our study found that polydrug use was substantial among PWID in Kohtla-Järve and St Petersburg, 44% of whom belonged to one of four polydrug classes. It also uncovered considerable differences in HIV risk behaviours with significantly greater injecting and sexual risk among polydrug than single-drug injectors (Table 4). Despite non-significant differences in HIV and HCV prevalence, riskier behaviours found among polydrug injectors suggest increased potential for continuing transmission of blood borne and sexually transmitted infections. Among polydrug classes, *polydrug*-*polyroute injectors* engaged in more frequent injecting and sharing risk behaviours than exclusive injectors. However, *opiate*-*stimulant poly*-*injectors* and *opiate*-*opioid poly*-*injectors* both reported more injecting and sexual risk behaviours than single drug injectors, with opiate-opioid poly-injectors also reporting sex partners who injected drugs. *Non*-*injection stimulant co*-*users* differed less from single-drug injectors but were more likely to back-load syringes and have had sex in the last six months (Table 4).

**Table 4:** Summary of associations between demographic, injecting and sexual risk behaviours and latent poly(drug) use classes as compared to single drug injectors

Class 1 Polydrug polyroute injection	Class 2 Opiate-stimulant poly-injection	Class 3 Non-injection stimulant co-use	Class 4 Opiate-opioid poly-injection
–	↑ <30 years old	↑ <30 years old	↑ <30 years old
↑ non- Russian ethnicity	–	–	↑ non- Russian ethnicity
–	–	↓ Secondary education	–
–	–	↑ Non-regular income	–
–	–	–	–
↑ Kohtla-Järve	–	↑ Kohtla-Järve	↑ St Petersburg
↑ Frequent injecting	↑ Frequent injecting	–	=Frequent injecting
↑ Intense injecting	↑ Intense injecting	–	↑ Intense injecting
↑ Shared needles/syringes	↑ Shared needles/syringes	–	↑ Shared needles/syringes
↑ Sharing paraphernalia	↑ Sharing paraphernalia	–	–
↑ Back-loaded	↑ Back-loaded	↑ Back-loaded	–
–	↑ Any sex last 6 months	↑ Any sex last 6 months	–
–	↑ Multiple sex partners	–	↑ Multiple sex partners
–	–	–	↑ Regular sex partner injects
–	–	–	↑ Casual sex partner injects

“↑and ↓” indicate positive and negative associations, respectively. “=” positive direction but non-significant association

Our findings that *opiate*-*stimulant poly*-*injection* was associated with frequent and intense injecting, needle/syringe sharing and multiple sex partners were consistent with several studies where PWID injecting opiates *and* stimulants (methamphetamines or cocaine) were more likely to report greater injection risks (i.e. sharing and lending needles/syringes [41, 63–66] and more daily injections [39–41, 66–69]) than single opiate injectors [40, 54, 68].

That *opiate*-*opioid poly*-*injection,* more likely to be found in St Petersburg, was associated with more daily injections and needle/syringe sharing in our study, likely reflected the localized availability and co-injection of illicit methadone and heroin in this city [45]. Injecting synthetic opioids and heroin has been associated with greater injection risks in some settings, including needle/syringe-sharing and more injections per day [70–73].

The following strengths and limitations are acknowledged. The samples may not be representative of all PWID in the two cities as they relied on a chain referral sampling methodology. RDS has nevertheless been an effective method to recruit hard-to-reach sub-groups, within key populations at risk of HIV infection, not reached by programmes [74].

Possible under-reporting of stigmatised behaviours in PWID self-reports and bias towards socially desirable answers were minimised by ensuring confidentiality during face-to-face interviews, in settings that were safe and familiar to PWID. Further, comparisons of missing data revealed no differences between classes suggesting that if bias due to non-response were present, it would affect polydrug sub-groups similarly.

It is also possible that short-term drug use patterns measured over four-weeks may not be a good predictor of HIV and HCV prevalence as polydrug class membership (and their associated behaviours) may not necessarily be longitudinally consistent or may not reflect cumulative exposure to certain risk factors. This may explain that, despite sexual and injecting risk behaviours being associated with polydrug use classes, we did not detect any association with HIV or HCV prevalence. Longitudinal studies among PWID may contribute to establish whether polydrug practices evolve over time, and especially how they might change over the course of a drug injection “career”, or as a function of the drug environment [53, 75].

The strengths of our analyses were the large sample size and combination of two similar samples of PWID, surveyed using comparable methods, instruments and tools, which could increase the generalisability of our results. Although the drug use and HIV epidemics were similar enough for surveys to be pooled, potential unobserved differences and within-survey correlations were possible and accounted for in the analysis. Our examination of effect modifications suggested that associations between polydrug use and risk behaviours were similar for both cities though the magnitude of some associations was greater for St Petersburg than Kohtla-Järve. Important structural differences in the two cities may also account for such differences. For instance, parenteral and oral routes of opioid use may co-occur in Kohtla-Järve, where oral methadone treatment is available but often at low doses; opioid highs may be sought by injecting locally available (methyl)-fentanyl. In St. Petersburg, however, heroin and illicit methadone are both injected and likely contribute most cases of opioid poly-injection.

The sizeable heterogeneity in polydrug use patterns and HIV risk behaviours among PWID highlight the need to expand opiate substitution treatment following evidence-based dosage guidelines at the public health scale. HIV prevention and treatment interventions need to be tailored to the risk profiles and drug combinations injected by PWID. Our findings also emphasize the importance of on-going and continuous drug monitoring among PWID, among whom polydrug use is frequent and may provide a marker of risk behaviours and HIV/HCV risk [76].

In these settings, HIV incidence among PWID was as high as 14.1 (95% CI 10.7–17.6) HIV infections per 100 person-years in St Petersburg in 2008 and 9 HIV infections per 100 person-years in Tallinn in 2009 [6, 9, 13, 77, 78]. Monitoring drugs and combinations commonly used by PWID could enable programmes to deliver appropriate injecting and sexual risk reduction messages, sufficient supplies of clean injection equipment and promotion of safe sexual behaviours *with all sex partners.* Whereas opiate substitution modalities should be expanded (or legalised in the case of Russia) to reduce risk among *opiate*-*opioid poly*-*injectors*, alternative drug treatment modalities are needed for *opiate*-*stimulant poly*-*injectors*. In settings where stimulant and opiates are injected, opiate substitution may not be effective and drug treatment alternatives including behavioural and pharmacological approaches for stimulant users are needed [27, 40, 79, 80]. Improving access to primary health care services should also be encouraged to provide an entry point for PWID with different drug use and risk profiles. Recognising and addressing polydrug use as drug combinations, in drug treatment and HIV treatment settings may also help to increase PWID retention, adherence to therapy and hence, improve treatment outcomes [24, 28, 81, 82].

## Electronic supplementary material

Below is the link to the electronic supplementary material.

Supplementary material 1 (DOCX 215 kb)
