# Supplementary material for: Polydrug Use and Heterogeneity in HIV Risk Among People Who Inject Drugs in Estonia and Russia: A Latent Class Analysis
Source: AIDS Behav. 2017 Jul 11;22(4):1329–40. doi: 10.1007/s10461-017-1836-0 (PMC5878835; doi:10.1007/s10461-017-1836-0)
Supplement: Supplementary file 1 — Supplementary material 1 (DOCX 215 kb) [file 10461_2017_1836_MOESM1_ESM.docx]

**Supplementary material:**

**Figures and Tables**

**Polydrug use and heterogeneity in HIV risk among people who inject drugs in Estonia and Russia: a latent class analysis**

**Authors:** Isabel Tavitian-Exley^1*^; Marie-Claude Boily^1^; Robert Heimer^2^; Anneli uusküla^3^; Olga LEVINA^4^, Mathieu Maheu-Giroux^5^

^1^ Department of Infectious Disease Epidemiology, Imperial College London, London, United Kingdom.

^2^ Epidemiology of Microbial Diseases, School of Public Health, Yale University, New Haven, United States.

^3^ Faculty of Medicine, University of Tartu, Tartu, Estonia.

^4^ NGO Stellit, St Petersburg, Russian Federation.

^5^ Department of Epidemiology, Biostatistics and Occupational Health, McGill University, Montréal, Canada.

*Corresponding Author:

Tavitianexley@gmail.com;

Norfolk place,

London W2 1PG,

United Kingdom.

+44 7952622592

+95 9 976236235

+95 9 73207491

Submission category: Original research article

Declaration of interest: None declared.

Running head: Polydrug use and HIV risk behaviours

Figure S.1: Schematic representation of latent and observed variables used in latent class analysis

Note Figure S.1: The variable in the oval shape is the latent variable (polydrug use) and those in the rectangles denote the seven observed variables described in the methods. “Add.” indicates additional drug(s) injected and/or used by PWID in addition to their main/ primary drug. The “city” variable was not included alongside the seven variables forming the latent classes but was included as a covariate to adjust for potential effects in LCA.

Table S.1: PWID characteristics in Kohtla-Järve and St Petersburg: crude and RDS-adjusted estimates

|  | **Kohtla-Järve**  (n=591) | | | **St Petersburg** (n=811) | | |
| --- | --- | --- | --- | --- | --- | --- |
| **Variables** | **Crude estimates (95% CI)** ^(1)^ | **RDS II-adjusted**^(2)^**estimates (95% CI)** | **n** 591 | **Crude estimates (95% CI)** | **RDS II-adjusted**^(2)^**estimates (95% CI)** | **n** 811 |
| **Demographic characteristics** | |  |  |  |  |  |
| **Sex**^[missing KJ=2]^ |  |  |  |  |  |  |
| Male | 74% (66%-80%) | 72% (67%-78%) | 434 | 78% (74%-82%) | 77% (73%-80%) | 631 |
| Female | 26% (19%-34% | 27% (23%-33%) | 155 | 22% (18%-26%) | 23% (20%-27%) | 180 |
| **Age group**^[missing KJ=0]^ |  |  |  |  |  |  |
| < 30 years | 50% (46%-55%) | 48% (42%-55%) | 294 | 30% (26%-34%) | 29% (25% - 33%) | 241 |
| >= 30 years | 50% (47%-53%) | 51% (45%-58%) | 297 | 70% (66%-74%) | 71% (67% - 74%) | 570 |
| **Ethnicity**^[missing KJ=1]^ |  |  |  |  |  |  |
| Russian | 81% (78%-85%) | 81% (76%-86%) | 481 | 96% (92%-98%) | 95% (93% - 97%) | 775 |
| Estonian | 12% (9%-13%) | 12% (7%-16%) | 66 | - | - | 0 |
| Non-Russian | 7% (5%-10%) | 7.1% (4%-10%) | 43 | 4% (2%-8%) | 5% (3% - 6%) | 36 |
| **Education level**^[missing KJ=0]^ | |  |  |  |  |  |
| Basic/Vocational | 80% (74%-84%) | 80% (75%-85%) | 472 | 58% (55%-62%) | 58% (53% - 62%) | 475 |
| Secondary | 19% (15%-25%) | 19% (15%-24%) | 116 | 30.0% (27%-33%) | 28% (26% - 31%) | 243 |
| Higher | 1% (0.5%-2%) | 1% (0.5%-3%) | 3 | 12% (9%-14%) | 14% (9% - 18%) | 93 |
| **Drug use characteristics**(last 4 weeks) | |  |  |  |  |  |
| **Main drug injected**^[missing KJ=2]^ | |  |  |  |  |  |
| Opiates | 61% (57%-65%) | 52% (45%-58%) | 362 | 96% (93%-98%) | 96% (94% - 98%) | 784 |
| ATS stimulants | 33% (29%-38%) | 41% (33%-48%) | 195 | 4% (1%-7%) | 4% (1% - 5%) | 27 |
| Other | 6% (4%-8%) | 7% (2%-12%) | 33 | 0.0% | 0.0% | 0 |
| **Polydrug use**^[missing KJ=0]^ |  |  |  |  |  |  |
| Any polydrug use | 47% (44%-50%) | 43% (37%-50%) | 277 | 41% (35%-48%) | 44% (39%-48%) | 335 |
| Single drug | 53% (50%-56%) | 57% (50%-63%) | 314 | 59% (52%-65%) | 56% (52%-61%) | 476 |
| **Poly-injection** |  |  |  |  |  |  |
| Polydrug injecting | 20% (17%-23%) | 26% (19%-32%) | 117 | 40% (33%-47%) | 42% (37%-46%) | 321 |
| Single drug | 80% (77%-83%) | 74% (68%-81%) | 474 | 60% (53%-67%) | 58% (54%-63%) | 490 |
| **Non-injection polydrug use** | |  |  |  |  |  |
| Non-injection polydrug | 41% (38%-44%) | 47% (42%-55%) | 242 | 7% (5%-10%) | 7% (5%-9%) | 56 |
| Single drug | 59% (56%-62%) | 53% (45%-58%) | 349 | 93% (90%-95%) | 93% (91%-95%) | 755 |
| **Other opiate injected** |  |  |  |  |  |  |
| Other opiate injected | 6% (4%-9%) | 7% (3%-11%) | 32 | 35% (28%-42%) | 37% (32%-41%) | 278 |
| No other opiate | 94% (91%-96%) | 93% (88%-97% | 494 | 65% (58%-72%) | 63% (59%-68%) | 515 |
| **Other stimulant injected** | |  |  |  |  |  |
| Other stimulant | 16% (13%-20%) | 22% (16%-28%) | 95 | 14% (10%-18%) | 15% (11%-18%) | 109 |
| No other stimulant | 84% (80%-87%) | 78% (72%-83.9%) | 486 | 86% (82%-90%) | 86% (82%-89%) | 695 |
| **Other opiate used** |  |  |  |  |  |  |
| Other opiate used | 12% (10%-15%) | 18% (12%-24%) | 65 | 6% (4%-8%) | 6.2% (4%-8%) | 46 |
| No other opiate | 88% (85%-90%) | 82% (76%-88%) | 462 | 94% (92%-96%) | 93.8% (92%-96%) | 757 |
| **Other stimulant used** |  |  |  |  |  |  |
| Other stimulant used | 39% (35%-42%) | 49% (43%-57%) | 228 | 3% (2%-6%) | 3% (2%-4%) | 27 |
| No other stimulant | 61% (58%-65%) | 51% (43%-57%) | 360 | 97% (94%-98%) | 97% (96%-99%) | 767 |
| **Contact with harm reduction interventions** | |  |  |  |  |  |
| **Drug treatment**(ever)^[missing KJ=0]^ | |  |  |  |  |  |
| Ever had treatment | 55% (50%-59%) | 57% (51%-64%) | 324 | 72% (67%-76%) | 71% (66%-74%) | 582 |
| Never had treatment | 45% (40%-50%) | 43% (36%-50%) | 267 | 28% (24%-33%) | 29% (25%-33%) | 229 |
| **Drug/substitution treatment**(12 months)^[missing KJ=0]^ | | |  |  |  |  |
| Yes | 13% (10%-16%) | 9% (6%-11%) | 75 | 11% (7%-15%) | 11% (8%-13%) | 86 |
| No | 87% (84%-90%) | 91% (87%-94%) | 516 | 89% (85%-93%) | 89% (86%-92%) | 724 |
| **Contact with NSP**(6 weeks)^[missing KJ=38]^ | |  |  |  |  |  |
| NSP | 82% (78%-85%) | 76% (70%-83%) | 451 | 16% (11%-21%) | 15% (12%-18%) | 119 |
| No NSP | 18% (16%-22%) | 24% (17%-30%) | 102 | 84% (79%-88%) | 85% (81%-88%) | 645 |
| **Serological markers** |  |  |  |  |  |  |
| **HIV status**^[missing KJ=0; SP=0]^ |  |  |  |  |  |  |
| Positive | 61% (56%-67%) | 52% (45%-59%) | 366 | 56% (51%-60%) | 55% (50%- 59%) | 452 |
| Negative | 39% (32%-44%) | 48% (41%-55%) | 225 | 44% (39%-48%) | 45% (41%- 49%) | 359 |
| **HCV status** ^[missing KJ=0]^ |  |  |  |  |  |  |
| Reactive | 75% (69%-80%) | 69% (62%-75%) | 441 | Not collected | Not collected | - |
| Non-reactive | 25% (20%-31%) | 31% (25%-38%) | 150 |  |  |  |
| **HSV status** ^[missing KJ=15)^ |  |  |  |  |  |  |
| Positive | 32% (26%-39%) | 34% (27%-39%) | 185 | Not collected | Not collected | - |
| Negative | 68% (61%-74%) | 66% (59%-71%) | 391 |  |  |  |

Table S1: (1)Column percentage. (2)Results are presented for crude estimates and RDS II (Volz-Heckathorn)-adjusted estimates for Kohtla-Järve and St Petersburg. Estimates for Kohtla-Järve were run in RDS Package for R, using recruiter id. Population size estimate used n=4,000. Results for St Petersburg were run in RDS Analyst using the coupon method. Population size estimate N=83,118 (range: 77,320 - 88920). RDS=Respondent Driven Sampling; CI=Confidence Intervals; ATS= Amphetamine-Type Stimulants. HIV= Human Immune deficiency Virus. The number of missing observations, if any, is indicated as follows for Kohtla-Järve^[missing KJ=n1]^and St Petersburg^[missing SP=n2]^, respectively.

Table S.2: Key respondent driven sampling survey characteristics and diagnostic measures (number of seeds and waves, recruitment homophily and network size)

| **RDS diagnostic measures** | **Kohtla-Järve** | **St Petersburg** |
| --- | --- | --- |
| **Seed number** | 6 | 16 |
| **Waves** | 11 | 12 |
| **Recruitment homophily** |  |  |
| sex | 1.08 | 1.00 |
| ethnicity | 1.03 | 1.01 |
| main income | 1.05 | 0.99 |
| HIV status | 1.25 | 0.99 |
| any polydrug use | 1.01 | 1.07 |
| **Median network size** | 10 (IQR: 6-12) | 15 (IQR: 9-20) |

Table S2: RDS diagnostic measures and weighted estimates were generated using RDS package for R v.0.7-3 and RDS Analyst v.0.42. Homophily was defined as “the ratio of the number of recruits with similar characteristic as their recruiter, to the number expected if there was no homophily on the characteristic. A measure of homophily for HIV status close to +1 indicated little or no preferential recruitment on this characteristic and suggested that the recruitment was similar to what would have been expected by chance” (RDS package for R). RDS= Respondent Driven Sampling; IQR= Inter quartile range; HIV= Human Immune deficiency Virus.

Figure S.2. Convergence plots for HIV status in Kohtla-Järve and St Petersburg (RDSII estimator)

Convergence plots presented were generated using RDS II (Volz-Heckathorn) estimator for Kohtla-Järve and St Petersburg. Plots for Kohtla-Järve were run in RDS Package for R, using recruiter id and for St Petersburg in RDS Analyst using the coupon method.

Table S.3: Fit statistics and indices for a latent class analyses of seven indicators among people who inject drugs

| **Fit statistic** | **C2 Model** | | **C3 Model** | | **C4 Model** | | **C5 Model** | | **C6 Model** | |
| --- | --- | --- | --- | --- | --- | --- | --- | --- | --- | --- |
| **AIC** | 8413.9 |  | 7100.3 |  | 6388.1 |  | 6163.6 |  | 6035.8 |  |
| **BIC** | 8581.8 |  | 7357.3 |  | 6734.4 |  | 6598.9 |  | 6560.2 |  |
| **Pearson’s X2** | <0.0001 |  | <0.0001 |  | <0.0001 |  | <0.0001 |  | 0.0497 |  |
| **LR test X2** | <0.0001 |  | <0.0001 |  | <0.0001 |  | <0.0001 |  | 0.8714 |  |
| **LL** | -4174.9 |  | -3501.1 |  | -3128.1 |  | -2998.8 |  | -2917.8 |  |
| **Entropy** | 1.000 |  | 0.998 |  | 1.000 |  | 1.000 |  | 1.000 |  |

Table S.3 notes on model fit statistic: Latent class model with binary, categorical, nominal variables, no thresholds. Model set and replicated at 400 random starts. CZ= Z-class model. AIC= Akaike information criteria. BIC= and Bayesian information criteria. Test p-values shown for Pearson’s X2, Likelihood ratio (LR) and Lo-Mendell-Rubin (LMRT) which compares current n class to n-1 class model. LL=Log Likelihood. LL was not replicated in 6- class model.

**Interpretation of latent class analysis model fit statistics:**

A non-significant value for the LMRT suggested that the model with one fewer class better explained the data and a smaller AIC and BIC indicated a better model fit (1-3). *Entropy* provides a measure of the degree to which latent classes are distinct from each other, by estimating individual conditional probabilities of class membership to assess the precision of class assignment and thus the usefulness and value of the resulting classes. Preference was given to entropy statistic values closest to +1 indicating greater entropy (1, 3). Individuals within a given class or sub-type were considered homogenous when they had similar item responses and when class-specific response probabilities for binary indicators were above 0.70 or below 0.30 (1). Models were estimated using maximum likelihood with a minimum of 400 random starts to ensure that global maxima solutions were reached (4). Conditional probabilities are the posterior probabilities of endorsing a drug variable for an individual classified in their most likely class in the five-class model.

Table S.4: Adjusted multinomial regression models with effect modification by city

|  | Class 1 (n=124) | Class 2 (n=97) | Class 3 (n=174) | Class 4 (n=219) | **Obs** |
| --- | --- | --- | --- | --- | --- |
| **Injecting risk behaviours** | **Polydrug polyroute injection** | **Opiate-stimulant poly-injection** | **Non-injection stimulant co-use** | **Opiate-opioid poly-injection** | n |
| **St Petersburg** |  |  |  |  |  |
| Injecting < 5 years | 1.1 (0.7-1.9) | 1.6 (0.4-6.3) | 0.7 (0.4-1.3) | 1.8 (0.5-5.9) | 1355 |
| Injected daily or more | 12.5 (4.1-37.4)* | 5.8 (3.3-10.2)* | 1.3 (0.3-4.9) | 1.7 (1.3-2.2)* | 1308 |
| Injected ≥ twice a day | 24.9 (8.1-76.6) | 5.6 (2.9-10.6) | 2.4 (0.8-7.8) | 2.0 (1.3-3.0) | 1305 |
| Shared needles/syringes | 2.5 (1.2-5.3) | 2.5 (1.8-3.7) | 0.7 (0.2-2.3) | 1.5 (1.1-2.5) | 1296 |
| Lent needles/syringes | 4.5 (2.3-8.7)* | 3.4 (2.1-5.4)* | 0.6 (0.2-1.8) | 1.5 (1.1-2.2) | 1303 |
| Shared paraphernalia | 2.9 (1.5-5.7) | 1.8 (1.1-2.4) | 0.7 (0.4-2.6) | 1.2 (0.8-1.7) | 1307 |
| Filled from working syringe | 3.8 (2.3-16.1) | 1.6 (1.0-2.7) | 1.0 (0.3-3.1) | 0.7 (0.4-1.1) | 1306 |
| **Kohtla-Järve** |  |  |  |  |  |
| Injecting < 5 years | 1.1 (0.7-1.9) | 1.6 (0.4-6.3) | 0.7 (0.4-1.3) | 1.8 (0.5-5.9) | 1355 |
| Injected daily or more | 0.8 (0.5-1.4)* | 0.4 (0.2-1.0)* | 0.6 (0.4-0.8) | 0.5 (0.2-1.3) | 1308 |
| Injected ≥ twice a day | 1.0 (0.6-1.8) | 2.0 (1.0-4.0) | 0.8 (0.7-1.1) | 1.6 (0.5-5.2) | 1305 |
| Shared needles/syringes | 3.1 (1.1-8.8) | 0 cell KJ | 2.8 (1.1-6.9) | 6.2 (1.3-30.2) | 1296 |
| Lent needles/syringes | 0.4 (0.3-0.6)* | 0.5 (0.1-2.9)* | 0.8 (0.4-1.7) | 1.0 (0.3-3.0) | 1303 |
| Shared paraphernalia | 3.6 (1.1-12.3) | 3.3 (0.4-27.2) | 4.7 (1.7-13.3) | 0 cell KJ | 1307 |
| Filled from working syringe | 6.8 (2.3-20.7) | 7.0 (0.5-106) | 9.0 (3.2-25.3) | 0 cell KJ | 1306 |

Table S.4 notes: Multivariate multinomial regression models were adjusted for age, sex, education, income, ethnicity, contact with needle and syringe programme and city, with effect modification between risk behaviours and city: city*injecting risk behaviour. *Effect modification p-value ≤0.05 in multinomial regression for cities combined using Kohtla-Järve as reference, then St Petersburg. †Confidence Intervals.

**References**

1. Roesch SC, Villodas M, Villodas F. Latent class/profile analysis in maltreatment research: A commentary on Nooner et al., Pears et al., and looking beyond. Child Abuse Negl. 2010;34(3):155-60.

2. Lanza ST, Bray BC, Collins LM. An Introduction to Latent class and Latent transition analysis. 2013. In: Handbook of Psychology, Vol 2: Research Methods in Psychology [Internet]. Hoboken, NJ, US: Wiley. 2nd. [691-715].

3. Flaherty BP, Kiff CJ. Latent class and latent profile models. In: Cooper H, Camic PM, Long DL, Panter AT, Rindskopf D, Sher KJ, editors. APA handbook of research methods in psychology, Vol 3: Data analysis and research publication. Washington, DC, US: American Psychological Association; 2012. p. 391-404.

4. Muthén LK, Muthén BO. Mplus User’s Guide. . Los Angeles, CA: Muthén & Muthén; 1998-2012.
